# “IT’S NOT THE DESTINATION BUT THE JOURNEY”: PERSPECTIVES AND ADVICE ON WISDOM

**DOI:** 10.1093/geroni/igad104.2318

**Published:** 2023-12-21

**Authors:** Nicole Lehpamer

**Affiliations:** Mather Institute, Evanston, Illinois, United States

## Abstract

While wisdom has been a common topic of contemplation for hundreds of years, a common theme in the wisdom literature relates to how wisdom is acquired through experiences. However, others have found that it can also be developed through individual factors, such as cognition, emotional intelligence, and personality, and cultivated through social interactions and self-reflection. Older adults have more experience than those of any other generation, giving them the time to develop the individual factors associated with wisdom and the skills to cultivate it. Given their unique social position, older adults are thus most qualified to share wisdom. In attempts to capitalize on the wisdom of older adults, the study uses survey data from adults aged 55 and better to examine how older adults characterize wisdom, it’s association with age, and how it can be achieved. Data was collected using surveys administered through the Survey Monkey Platform and the Mather Institute Research Panel. Findings indicate that most older adults believe that wisdom increases with age. They further believe that wise individuals accept their limitations, are realists, have developed strong relationships with others, and seem in control of their lives. Older adults believe that wisdom can primarily be achieved by making mistakes, interacting with people who are different, experiencing adversity, and trying new things. Understanding the ways in which older adults perceive wisdom can be used to enhance intergenerational interactions that are mutually beneficial.

